# Smoking and prevalence of allergic disorders in Japanese pregnant women: baseline data from the Kyushu Okinawa Maternal and Child Health Study

**DOI:** 10.1186/1476-069X-11-15

**Published:** 2012-03-14

**Authors:** Keiko Tanaka, Yoshihiro Miyake, Masashi Arakawa

**Affiliations:** 1 Department of Preventive Medicine and Public Health, Faculty of Medicine, Fukuoka University, Fukuoka 814-0180, Japan; 2 Course of Wellness, Graduate School of Tourism Sciences, University of the Ryukyus, Okinawa, Japan

**Keywords:** Asthma, Cross-sectional studies, Eczema, Environmental tobacco smoke, Smoking, Wheeze, Rhinoconjunctivitis

## Abstract

**Background:**

Studies on the associations between smoking and allergic diseases have mostly focused on asthma. Epidemiological studies in adults on the effects of smoking on allergic diseases other than asthma, such as eczema and rhinoconjunctivitis, have been limited, and the information that is available has been inconsistent. The aim of this study was to investigate the association between smoking status and environmental tobacco smoke (ETS) exposure and the prevalence of allergic diseases.

**Methods:**

Study subjects were 1743 pregnant Japanese women. The definitions of wheeze and asthma were based on criteria from the European Community Respiratory Health Survey whereas those of eczema and rhinoconjunctivitis were based on criteria from the International Study of Asthma and Allergies in Childhood. Adjustment was made for age; region of residence; family history of asthma, atopic eczema, and allergic rhinitis; household income; and education.

**Results:**

Compared with never smoking, current smoking and ≥ 4 pack-years of smoking were independently positively associated with the prevalence of wheeze. There were no associations between smoking status and the prevalence of asthma, eczema, or rhinoconjunctivitis. When subjects who had never smoked were classified into four categories based on the source of ETS exposure (never, only at home, only at work, and both), exposure occurring both at home and at work was independently associated with an increased prevalence of two outcomes: wheeze and rhinoconjunctivitis. No relationships were observed between exposure to ETS and the prevalence of asthma or eczema.

**Conclusions:**

Our results provide evidence that current smoking and ETS exposure may increase the likelihood of wheeze. The possibility of a positive association between ETS exposure and rhinoconjunctivitis was also suggested.

## Introduction

Cigarette smoking is a known risk factor for various neurological, cardiovascular, and pulmonary diseases such as chronic obstructive pulmonary disease and lung cancer [1]. Research on the association between smoking and allergic diseases has mostly focused on asthma, and most evidence suggests that smoking is positively associated with asthma [2-6]. A population-based cohort study in Japan has shown that smoking is significantly associated with an increased risk of asthma in men, though this significant positive association was not observed in women [2]. Moreover, in the National Health and Nutrition Examination (USA), no association was observed between current smoking and asthma [6]. Epidemiological studies in adults on the effects of smoking on allergic diseases other than asthma, such as eczema and rhinoconjunctivitis, have been limited [7-12].

Evidence of the adverse health effects of exposure to environmental tobacco smoke (ETS) has recently been accumulating. Most publications on ETS exposure have focused on respiratory symptoms and asthma among children. Evidence concerning the relationship between ETS and allergic diseases in adults has been limited and has provided contradictory results [13-21]. A prospective study among non-smoking young adults in Canada has shown no association between ETS exposure and the risk of wheeze [13]. In a cross-sectional study among never-smoking Italian women, on the other hand, exposure to ETS was positively associated with the prevalence of wheeze, asthma, and rhinoconjunctivitis [14]. Assessment of the results of exposure to ETS might be more complex in adults than in children, because adults are exposed to ETS from multiple sources, such as the home, the workplace, and public places [22].

In short, the literature on the association between smoking and allergic diseases in adults has yielded heterogeneous findings. In the present study, we investigated whether smoking status and exposure to ETS at home and at work was associated with a higher prevalence of wheeze, asthma, eczema, and rhinoconjunctivitis among pregnant Japanese women, using the baseline data set of the Kyushu Okinawa Maternal and Child Health Study (KOMCHS).

## Methods

### Study population

The KOMCHS is an ongoing prospective birth cohort study that investigates preventive and risk factors for maternal and child health problems such as allergic disorders. The background and general procedure of the KOMCHS have been described previously [23,24]. In brief, the KOMCHS requested that pregnant women complete a baseline survey, which was followed by several post-natal surveys. Eligible subjects were those women who became pregnant in one of seven prefectures on Kyushu Island in southern Japan or Okinawa Prefecture between April 2007 and March 2008. At 423 obstetric hospitals, a set of leaflets explaining the KOMCHS, an application form to participate in the study, and a self-addressed and stamped return envelope were distributed to pregnant women, insofar as this was possible. Pregnant women who intended to participate in the KOMCHS returned the application form to the data management center. In the end, a total of 1757 pregnant women between the 5th and 39th week of pregnancy gave their fully informed consent in writing to participate and completed the baseline survey. Excluded were 14 pregnant women because of missing data on the factors under study, leaving data on 1743 pregnant women available for analysis. The ethics committee of the Faculty of Medicine, Fukuoka University, approved the KOMCHS.

### Measurements

In the baseline survey, each participant filled out a set of 2 self-administered questionnaires. Participants mailed the answered questionnaires to the data management center. Research technicians completed missing or illogical data by telephone interview.

One of the self-administered questionnaires included questions on wheeze and asthma based on the European Community Respiratory Health Survey [25] and questions on eczema and rhinoconjunctivitis based on the International Study of Asthma and Allergies in Childhood [26,27]. Wheeze was defined as a positive response to the question, 'Have you had wheezing or whistling in your chest at any time in the last 12 months?' Asthma was defined as a positive response to either of 2 situations: an asthma attack during the last 12 months or current use of asthma medication. Affirmative answers to the following 3 questions were required to indicate the presence of eczema: 'Have you ever had an itchy rash which was coming and going for at least 6 months?', 'Have you had this itchy rash at any time in the last 12 months?' and 'Has this itchy rash at any time affected any of the following places: the folds of the elbows, behind the knees, in front of the ankles, under the buttocks, or around the neck, ears, or eyes?' Rhinoconjunctivitis was defined as present by a positive response to the following 2 questions: 'In the last 12 months, have you had a problem with sneezing or a runny or blocked nose when you did not have a cold or flu?' and 'In the last 12 months, has this nose problem been accompanied by itchy-watery eyes?' The questionnaire also elicited information on age, smoking habits, ETS exposure at home and at work, family history of asthma, atopic eczema, and allergic rhinitis, household income, and education. Cumulative exposure to cigarette smoking was summarized by multiplying the average number of packs smoked per day (cigarettes smoked per day divided by 20) by the number of years smoked (pack-years of smoking), regardless of whether smoking status was former or current. Exposure to ETS at home and at work was assessed by the questions "Have you ever been exposed to smoke from family members at home?" and "Have you ever been exposed to smoke at your workplace?" A family history of asthma, atopic eczema, or allergic rhinitis (including Japanese cedar pollinosis) was considered to be present if one or more parents or siblings of the study subjects had been diagnosed by a physician as having any of these allergic disorders.

The second questionnaire was a validated self-administered diet history questionnaire. Data regarding diet were not used in the present study.

### Statistical analysis

Age; region of residence; family history of asthma, atopic eczema, and allergic rhinitis; household income; and education were a priori selected as potential confounding factors. Region of residence was classified into 3 categories (Fukuoka Prefecture, other than Fukuoka Prefecture on Kyushu Island, and Okinawa Prefecture), household income into 3 (< 4,000,000, 4,000,000 - 5,999,999, and ≥ 6,000,000 yen/year), education into 3 (< 13, 13-14, and ≥ 15 years; these levels are equivalent to high school, junior college or vocational/technical school, and university or more, respectively), smoking history into 3 (never, former, and current), pack-years of smoking into 3 (none, 0.05-3.9, and ≥ 4.0), and lifetime ETS exposure into 4 (never, only at home, only at work, and both at home and at work). Age was used as a continuous variable. Multiple logistic regression analysis was performed to estimate adjusted odds ratios (ORs) and 95% confidence intervals (CIs) of allergic disorders relative to smoking status. Trend of association was assessed by a logistic regression model assigning consecutive integers to the categories of the exposure variables. All statistical analyses were performed using the SAS software package version 9.2 (SAS Institute, Inc., Cary, NC, USA).

## Results

The prevalence values of wheeze, asthma, eczema, and rhinoconjunctivitis in the past 12 months were 10.4%, 5.5%, 13.0%, and 25.9%, respectively, among the 1743 pregnant women. Characteristics of the study subjects are described in Table 1. The mean age of the participants at baseline were 31.2 years. Many more participants had a family history of allergic rhinitis than a family history of asthma or atopic eczema.

**Table 1:** Distribution of selected characteristics in 1743 pregnant women, Kyushu Okinawa Maternal and Child Health Study, Japan

Variable	No. (%) or mean ± SD
Age, years, mean ± SD	31.2 ± 4.4
Region of residence, %	
Fukuoka Prefecture	970 (55.7)
Other than Fukuoka Prefecture in Kyushu	591 (33.9)
Okinawa Prefecture	182 (10.4)
Family history of asthma, %	330 (18.9)
Family history of atopic eczema, %	302 (17.3)
Family history of allergic rhinitis, %	759 (43.6)
Household income, %, yen/year	
< 4,000,000	632 (36.3)
4,000,000 - 5,999,999	618 (35.5)
≥ 6,000,000	493 (28.3)
Education, %, years	
< 13	428 (24.6)
13 - 14	576 (33.1)
≥ 15	739 (42.4)
Smoking status, %	
Never	1182 (67.8)
Former	503 (28.9)
Current	58 (3.3)
Pack-years of smoking, %	
None	1182 (67.8)
0.05 - 3.9	262 (15.0)
≥ 4.0	299 (17.2)

The prevalence of allergic diseases according to smoking status is provided in Table 2. There is a stepwise increase in the prevalence of wheeze and asthma in relation to smoking status; prevalence is highest among current smokers. Table 3 presents the prevalence of allergic diseases according to ETS exposure status among 1182 pregnant women who had never smoked. For all allergic diseases, the highest prevalence was observed in women who were exposed to ETS both at home and at work; the prevalence values of wheeze, asthma, eczema, and rhinoconjunctivitis among women in this category were 11.6%, 6.6%, 13.3%, and 28.2%, respectively.

**Table 2:** Prevalence of allergic disorders according to smoking status in 1743 pregnant women, Kyushu Okinawa Maternal and Child Health Study, Japan

		Wheeze		Asthma		Eczema		Rhinoconjunctivitis	
					
	N	n	%	n	%	n	%	n	%
**Smoking status**									
Never	1182	105	8.9%	58	4.9%	146	12.4%	302	25.6%
Former	503	65	12.9%	31	6.2%	72	14.3%	133	26.4%
Current	58	12	20.7%	7	12.1%	8	13.8%	17	29.3%
**Pack-years of smoking**									
None	1182	105	8.9%	58	4.9%	146	12.4%	302	25.6%
0.05-3.9	262	27	10.3%	11	4.2%	36	13.7%	68	26.0%
≥ 4.0	299	50	16.7%	27	9.0%	44	14.7%	82	27.4%

**Table 3:** Prevalence of allergic disorders according to ETS exposure status in 1182 pregnant women who had never smoked, Kyushu Okinawa Maternal and Child Health Study, Japan

		Wheeze		Asthma		Eczema		Rhinoconjunctivitis	
					
	N	n	%	n	%	n	%	n	%
**Lifetime ETS exposure at home or at work**									
Never	189	9	4.8%	6	3.2%	18	9.5%	37	19.6%
Only at home	313	26	8.3%	11	3.5%	39	12.5%	85	27.2%
Only at work	162	10	6.2%	7	4.3%	20	12.4%	34	21.0%
Both	518	60	11.6%	34	6.6%	69	13.3%	146	28.2%

Table 4 shows ORs and 95% CIs for allergic disorders in relation to smoking status. Compared with never smoking, current smoking was positively associated with the prevalence of wheeze: adjusted OR was 1.52 (95% CI: 1.05 to 2.12). There was no statistically significant association between former smoking and the prevalence of wheeze. Compared with never smoking, ≥ 4 pack-years of smoking was significantly positively associated with wheeze, showing a clear dose-response relationship with the cumulative consumption of cigarettes (*P *for linear trend = 0.002). There were no associations between smoking status and the prevalence of asthma, eczema, or rhinoconjunctivitis.

**Table 4:** Crude and adjusted odds ratios (ORs) and 95% confidence intervals (CIs) for allergic disorders according to smoking status in 1743 pregnant women, Kyushu Okinawa Maternal and Child Health Study, Japan

	Wheeze		Asthma		Eczema		Rhinoconjunctivitis	
				
	Crude OR (95% CI)	Adjusted OR^1 ^(95% CI)	Crude OR (95% CI)	Adjusted OR^1 ^(95% CI)	Crude OR (95% CI)	Adjusted OR^1 ^(95% CI)	Crude OR (95% CI)	Adjusted OR^1 ^(95% CI)
**Smoking status**								
Never	1.00	1.00	1.00	1.00	1.00	1.00	1.00	1.00
Former	1.52 (1.09-2.11)	1.39 (0.98-1.95)	1.27 (0.80-1.98)	1.07 (0.66-1.70)	1.19 (0.87-1.60)	1.16 (0.84-1.58)	1.05 (0.83-1.33)	0.99 (0.77-1.27)
Current	1.64 (1.15-2.25)	1.52 (1.05-2.12)	1.63 (1.03-2.40)	1.40 (0.87-2.10)	1.07 (0.70-1.52)	0.98 (0.64-1.42)	1.10 (0.81-1.46)	1.05 (0.77-1.41)
**Pack-years of smoking**								
None	1.00	1.00	1.00	1.00	1.00	1.00	1.00	1.00
0.05-3.9	1.18 (0.74-1.82)	1.03 (0.64-1.61)	0.85 (0.42-1.58)	0.71 (0.34-1.34)	1.13 (0.75-1.66)	1.02 (0.67-1.52)	1.02 (0.75-1.38)	0.92 (0.67-1.25)
≥ 4.0	2.06 (1.42-2.95)	1.93 (1.30-2.83)	1.92 (1.18-3.07)	1.58 (0.94-2.58)	1.22 (0.84-1.75)	1.25 (0.85-1.82)	1.10 (0.82-1.46)	1.08 (0.80-1.46)
*P *for trend	0.0002	0.002	0.02	0.15	0.25	0.29	0.52	0.75

^1 ^Adjustment for age; region of residence; family history of asthma, atopic eczema, and allergic rhinitis; household income; and education

Table 5 shows the association between exposure to ETS and allergic disorders among the 1182 subjects who had never smoked. When the subjects who had never smoked were classified into four mutually exclusive categories based on the source of ETS exposure (never, only at home, only at work, and both), exposure to ETS both at home and at work was independently associated with a 2.7-fold increased prevalence of wheeze (adjusted OR = 2.73, 95% CI: 1.37 to 6.08), while no association was observed between exposure to ETS only at home or only at work and wheeze. Exposure to ETS only at home and exposure to ETS both at home and at work were independently positively associated with rhinoconjunctivitis: adjusted ORs for only at home and for both at home and at work were 1.63 (95% CI: 1.05 to 2.57) and 1.69 (95% CI: 1.12 to 2.59), respectively. The association between exposure to ETS only at work and the prevalence of rhinoconjunctivitis was not significant. There were no relationships between exposure to ETS and the prevalence of asthma or eczema.

**Table 5:** Crude and adjusted odds ratios (ORs) and 95% confidence intervals (CIs) for allergic disorders according to ETS exposure status in 1182 pregnant women who had never smoked, Kyushu Okinawa Maternal and Child Health Study, Japan

	Wheeze		Asthma		Eczema		Rhinoconjunctivitis	
				
	Crude OR (95% CI)	**Adjusted OR**^**1 **^**(95% CI)**	Crude OR (95% CI)	Adjusted OR^1 ^(95% CI)	Crude OR (95% CI)	Adjusted OR^1 ^(95% CI)	Crude OR (95% CI)	Adjusted OR^1 ^(95% CI)
**Lifetime ETS exposure at home or at work**								
Never	1.00	1.00	1.00	1.00	1.00	1.00	1.00	1.00
Only at home	1.81 (0.86-4.17)	1.83 (0.86-4.25)	1.11 (0.42-3.27)	1.08 (0.40-3.20)	1.35 (0.76-2.49)	1.39 (0.77-2.59)	1.53 (1.00-2.39)	1.63 (1.05-2.57)
Only at work	1.32 (0.52-3.39)	1.24 (0.48-3.25)	1.38 (0.45-4.36)	1.18 (0.38-3.79)	1.34 (0.68-2.65)	1.29 (0.65-2.60)	1.09 (0.65-1.84)	1.10 (0.64-1.88)
Both	2.62 (1.34-5.76)	2.73 (1.37-6.08)	2.14 (0.95-5.75)	1.96 (0.85-5.34)	1.46 (0.86-2.59)	1.55 (0.90-2.80)	1.61 (1.08-2.45)	1.69 (1.12-2.59)

^1 ^Adjustment for age; region of residence; family history of asthma, atopic eczema, and allergic rhinitis; household income; and education

## Discussion

In this study, we found that current smoking and ≥ 4 pack-years of smoking were independently positively related to wheeze. These findings are in accordance with results from previous cross-sectional studies that reported a positive association between ever smoking and the prevalence of wheeze in adults [12,18]. A population-based cross-sectional study in Denmark showed a significantly positive dose-response relationship between the amount of tobacco used per day and the prevalence of wheeze [18]. In a cross-sectional study among male farmers, current smoking was associated with an increased prevalence of wheeze [12]. Between asthma and smoking status, on the other hand, we found no association. The relationship between smoking status and asthma in adults remains controversial because the results of previous studies have been inconclusive; some studies have found an increased risk of asthma in relation to smoking [2-5], and others have failed to find such a correlation [2,6]. In a population-based cohort study among men in Japan, current smokers at baseline had a significantly increased risk of asthma compared with those who had never smoked, though no such statistically significant association was observed among women [2]. A cross-sectional study of pregnant Japanese women showed that current smoking was associated with an increased prevalence of asthma after the age of 18 years [4]. In a US cross-sectional study, former smoking was positively associated with the prevalence of asthma among males, whereas no association was observed between current smoking and the prevalence of asthma [6]. Among females in the same study, however, there was no association between former or current smoking and the prevalence of asthma [6].

The lack of association between smoking status and asthma observed in the present study might be explained by the "healthy smoker effect" [28,29]. Current smoking might be associated with better health, as healthier individuals are better able to tolerate the negative health effects of smoking. Thus, current smokers may be less likely to have or develop asthma. Moreover, the fact that there were only 38 females with asthma who had ever smoked in our study might have led to low statistical power for detecting a possible association between ever smoking and asthma.

Previous studies on smoking and eczema and/or rhinoconjunctivitis in adults have been limited and have provided contradictory results. Our results regarding eczema are in agreement with those of a previously cited Japanese cross-sectional study demonstrating no association between cigarette smoking and atopic eczema [4]. A case-control study in Taiwan, in contrast, found that smoking was associated with increased risk of adult-onset atopic eczema [11]. No association between smoking status and rhinoconjunctivitis was found in the present study. The same conclusion has also been reached in some previous studies [4,10], while other previous studies have observed inverse associations between smoking and rhinitis, hay fever, or cedar pollinosis [7-9]. It should be noted that the various studies mentioned here have used different definitions of outcomes, study populations and designs, smoking exposure assessment methods, and confounding factors, thus limiting the feasibility of inter-study comparisons.

In keeping with other previous studies [14,15,17,18], we found that ETS exposure was associated with an increased prevalence of wheeze. A cross-sectional study in Denmark has shown that daily ETS exposure for 5 or more hours is positively associated with the prevalence of wheeze among those who had never smoked [18]. A positive association between exposure to ETS and the prevalence of positive history of wheezing was found among male police officers in Hong Kong [15]. In contrast, a prospective study of subjects aged 15 to 40 years of age who had never smoked showed no association between ETS exposure and the risk of wheeze [13].

In our study, we found that the positive association between wheeze and exposure to ETS was more evident when exposure occurred at home rather than at work. A cross-sectional study among Italian women who had never smoked, on the other hand, revealed that ETS exposure at work was significantly positively associated with wheeze, whereas there was no association between wheeze and exposure to ETS due to their husbands' smoking [14]. A cross-sectional study performed in Finland, Estonia, and Sweden also showed a significant positive association between ETS exposure outside of the home, but not at home, and the prevalence of wheeze [17]. These results are at variance with our findings. In Japan, a law that restricts smoking in public places came into effect in 2004. Since then, awareness about the effects of ETS has been increasing, and many workplaces now have smoking restrictions or bans. In recent years, exposure to ETS at work is probably becoming less common. In the present study, exposure to ETS both at home and at work yielded a higher OR for wheeze than did exposure in only one location. Subjects who are exposed in both places are likely to receive a higher level of ETS exposure compared to those who are exposed in only one place.

A limited number of studies have addressed the effect of ETS exposure on asthma in adults [14,16,17]. A population-based incident case-control study in Finland found that both workplace and home ETS exposure during the past 12 months were significantly related to an increased risk of new development of asthma in 21- to 63-year-old adults [16]. A significant positive association between ETS exposure and asthma has also been reported in other cross-sectional studies [14,17]. In contrast, our results failed to reveal a positive association between ETS exposure and asthma. This may be due to a tendency among patients with asthma to voluntarily avoid exposure to ETS. Also, family members in a household that includes persons with asthma and coworkers of persons with asthma may be more likely to stop or curtail their smoking. In addition, it may be difficult to detect a clear positive association between ETS exposure and asthma in a population containing few patients with asthma.

To our knowledge, there have been two epidemiological studies addressing the association between ETS exposure and eczema in adults [4,11]. A case-control study in Taiwan showed that exposure to ETS at home prior to 20 years of age was positively associated with an increased risk of adult-onset atopic eczema among persons who had never smoked. A previously cited cross-sectional study among pregnant Japanese women found no association between ETS exposure at home or at work and the prevalence of eczema [4]. The findings of the latter study are in agreement with our own.

We found that exposure to ETS only at home and both at home and at work, but not only at work, was associated with an increased prevalence of rhinoconjunctivitis. Consistent with our results, several previously mentioned cross-sectional studies have found positive associations between ETS exposure and rhinoconjunctivitis [4,14] or rhinitis symptoms [8]. A cross-sectional study among Italian women who had never smoked showed a positive association between exposure to smoke from their husbands' smoking and rhinoconjunctivitis; this association was more evident than the relationship between ETS at work and rhinoconjunctivitis [14]. Our results were at variance with those of other studies that have found no association between ETS exposure and rhinitis [20,21].

This study had certain methodological strengths. Study subjects were homogeneous in that all were pregnant, which likely reduces the potential for confounding resulting from unmeasured factors related to pregnancy. Definitions of wheeze and asthma were based on the questions in the European Community Respiratory Health Survey. The prevalence values for eczema and rhinoconjunctivitis were estimated based on the questions in the International Study of Asthma and Allergies in Childhood, although validation tests for these questions have not been performed for Japanese adults. Using a standardized methodology allows for comparisons with results from other epidemiological studies using the same methodology. Potential confounders were adjusted for with extensive data. However, it is possible that our results remain confounded by other potentially important factors, such as aeroallergens and air pollution.

There were certain limitations of this study as well. The participation rate could not be calculated because the exact number of eligible pregnant women who were provided with a set of leaflets explaining the KOMCHS, an application form, and a self-addressed and stamped return envelope by the 423 collaborating obstetric hospitals is not available. We were not able to assess the differences between participants and non-participants, because information on personal characteristics such as age, socioeconomic status, and history of allergic disorders among non-participants is not available. Our subjects were probably not a representative sample of Japanese women in the general population, given that all were pregnant women. In addition, educational levels in the current study population were higher than in the general population. According to the 2000 population census of Japan, the proportions of women aged 30 to 34 years in Fukuoka Prefecture with < 13, 13-14, ≥ 15, and an unknown number of years of education were 52.0%, 31.5%, 11.8%, and 4.8%, respectively [30]. The corresponding figures for the current study were 24.6%, 33.1%, 42.4%, and 0.0%, respectively. The present population might therefore have had a greater awareness about health than the general population. Nevertheless, cigarette-smoking status in our study population was likely to be similar to that in the general population. In the National Health and Nutrition Survey in Japan in 2007, the percentages of currently-smoking, formerly-smoking, and non-smoking women aged 30 to 39 years were 17.2%, 11.4%, and 71.4%, respectively, although data specific to pregnant women were not available [31]. The corresponding figures for the present subjects were 3.3%, 28.9%, and 67.8%, respectively. Many of the pregnant women in the current study may have given up smoking upon becoming pregnant, thus increasing the percentage of former smokers.

Other limitations may also have influenced the interpretation of the current results. As this study is cross-sectional, the temporal nature of the association between smoking status and ETS exposure and allergic diseases could not be examined. Assessment of exposure was based on the subjects' questionnaire responses and was not validated by objective measurements, such as salivary, serum, or hair cotinine levels. Using questionnaires may result in misclassification due to recall bias. If present, however, this bias would tend to reduce the observed association between smoking and allergic diseases. We did not measure exposure to ETS at social settings outside the home and work. Given the difficulties in accurately measuring ETS exposure, the association between ETS exposure and allergic diseases observed here is likely to be an underestimate.

The interface between allergy/immunology and pregnancy should be discussed, as it may have an influence on the association of interest. It has been suggested that pregnancy involves a shift to the Th2 side of the immune response [32], although Chaouat et al. have pointed out the importance of the role of natural killer cells and IL-12, IL-15, and IL-18 tripods in successful or failed pregnancies in humans beyond the Th1/Th2 paradigm [33]. The hormonal changes in pregnancy are often invoked to explain the apparent association between rhinitis symptoms and pregnancy. However, rhinitis ascribed solely to pregnancy may not be a distinct entity because most pregnant women do not have significant nasal symptoms [32].

## Conclusions

Current smoking and ≥ 4 pack-years of smoking were significantly associated with increased prevalence of wheeze, but not of asthma, eczema, or rhinoconjunctivitis, among pregnant Japanese women. Positive associations between exposure to ETS and the prevalence of wheeze and that of rhinoconjunctivitis were found in subjects who had never smoked.

Even though more evidence is needed to validate the association between smoking exposure and allergic diseases in adults, there are limited studies which have focused on this association. This study provides valuable evidence regarding the association between smoking exposure and allergic diseases in adults. To clarify the associations between current or former smoking or ETS exposure and allergic diseases among adults, further studies with longitudinal design and more precise measurements of exposure, such as salivary, serum, or hair cotinine levels, will be needed. A follow-up survey of the KOMCHS will allow us to establish whether smoking exposure is an important predictor of allergic disorders in adulthood.

## Abbreviations

CI: Confidence interval; ETS: Environmental tobacco smoke; KOMCHS: Kyushu Okinawa Maternal and Child Health Study; OR: Odds ratio

## Competing interests

The authors declare that they have no competing interests.

## Authors' contributions

All authors contributed to the study concept and design and the acquisition of data. KT was responsible for the analysis and interpretation of data and the drafting of the manuscript. All authors participated in critically revising the manuscript and approved the final version of the manuscript.
